# Sex differences in expression of immune elements emerge in children, young adults and mice with osteosarcoma

**DOI:** 10.1186/s13293-020-00347-y

**Published:** 2021-01-06

**Authors:** Lauren J. Mills, Logan G. Spector, David A. Largaespada, Lindsay A. Williams

**Affiliations:** 1 Department of Pediatrics, University of Minnesota School of Medicine, Minneapolis, MN USA; 2 Masonic Cancer Center, University of Minnesota, Minneapolis, MN USA; 3 Division of Epidemiology and Clinical Research, Department of Pediatrics, University of Minnesota, Minneapolis, MN USA; 4 Brain Tumor Program, University of Minnesota, Minneapolis, MN USA; 5 Department of Genetics, Cell Biology and Development, University of Minnesota School of Medicine, Minneapolis, MN USA; 6 Center for Genome Engineering, University of Minnesota, Minneapolis, MN USA

**Keywords:** Osteosarcoma, Sex differences, Gene expression, Survival disparities, Pediatric and young adult cancer, Mouse osteosarcoma

## Abstract

**Background:**

Males < 40 years old are more likely to be diagnosed with and die from osteosarcoma (OS). The underlying mechanisms may depend on sex differences in immune response.

**Methods:**

We used SEER data to estimate survival differences between males and females aged < 40 years at OS diagnosis. In NCI TARGET-OS cases, we determined sex differences in gene expression, conducted Gene Set Enrichment Analysis (GSEA), and applied the LM22 signature to identify biologic sex differences. We compared sex differences in gene expression profiles in TARGET-OS to those observed in *Sleeping Beauty* (SB) transposon mutagenesis accelerated *Trp53*^*R270H*^-mutant mouse-OS and healthy adult osteoblasts.

**Results:**

Males had worse 17-year overall survival than females (SEER *p* < 0.0001). From 87 TARGET-OS cases, we observed 1018 genes and 69 pathways that differed significantly by sex (adjusted *p* < 0.05). Pathway and gene lists overlapped with those from mice (*p* = 0.03) and healthy osteoblasts (*p* = 0.017), respectively. Pathways that differed significantly by sex were largely immune-based and included the PD-1/PD-L1 immunotherapy pathway. We observed sex differences in M2 macrophages (LM22; *p* = 0.056) and M1-M2 macrophage transition (GSEA; *p* = 0.037) in TARGET-OS. LM22 trends were similar in mice. Twenty-four genes differentially expressed by sex in TARGET-OS had existing cancer therapies.

**Conclusions:**

Sex differences in OS gene expression were similar across species and centered on immune pathways. Identified sex-specific therapeutic targets may improve outcomes in young individuals with OS.

## Introduction

Osteosarcoma (OS), a bone tumor that displays a bimodal peak in incidence during the teen and late adult years [1], has a male predominance in incidence [2]. In children < 20 years old, OS displays a male excess of 33% in incidence [2] that is hypothesized to depend on rapid growth accompanying changes in the hormonal milieu in males and females during their respective pubertal windows [2–5]. OS has dismal 5-year survival at 60-70% [6]. Males < 20 years old at diagnosis have worse survival than females and experience a 30% excess risk of death, which we found to be largely independent of stage of disease [7]. The biologic reasons underlying the male excess in death may depend on sex differences in tumor biology or response to treatment.

Investigations into the somatic landscape of pediatric OS have reported much lower mutation frequencies than adult tumors [8], shown complex karyotypes characterized by chromosome arms losses/gains [9], and found frequent chromothripsis [10]. These large structural changes make the OS genome highly unstable stalling the development of OS-specific therapies in recent decades [11]. Beyond gross chromosomal abnormalities, genomic studies have also found that OS frequently harbors *TP53* mutations [12], which echoes the high risk of OS among individuals with Li-Fraumeni syndrome [13, 14]. We and others have found that OS is characterized by low immune cell infiltrate, which is prognostic [15, 16]. However, rarely, if ever, is sex considered in these analyses, which precludes us from identifying etiologic or biologic mechanisms that lead to a male excess in OS incidence and death [1].

We previously reported worse survival after an OS diagnosis for males compared to females < 20 years of age at diagnosis [7]. Therefore, we hypothesized that sex differences in OS biology may be one measurable mechanism that underlies the male excess in death. As such, our aim was to evaluate sex differences in OS gene expression among children and young adults using the publicly available NCI Therapeutically Applicable Research to Generate Effective Treatments (TARGET) initiative data and place these findings in context to those observed in a genetically engineered mouse model [17] and healthy adult osteoblasts [18]. The use of the mouse model allows us to determine the utility of studying sex differences in vivo as amassing a cohort of pediatric and young adult OS patients is challenging given the rarity of OS. The use of healthy adult osteoblasts serves as a baseline measure of sex differences in osteoblast gene expression as osteoblasts are hypothesized to be a likely cell of origin for OS [19]. Finally, using the information on differential gene expression from the TARGET-OS cases, we sought to identify potential sex-specific therapeutic targets for future exploration as there have been few improvements in OS treatment and survival since the 1980s [20].

## Materials and methods

### Data

To estimate sex differences in overall survival in children and young adults, we obtained data from the Surveillance, Epidemiology, and End Results (SEER) 18 registries (2000-2016) as described elsewhere [7]. Cases had a first primary, microscopically confirmed malignant OS and were included based on age at diagnosis matching that in TARGET (0-39 years). OS was categorized using ICD-O-3 codes 9180-9183, 9185-9187, 9192-9194; excluding OS in Paget disease (9184; date accessed: April 30, 2020) [20].

The results published here for human OS are based upon data generated by the NCI TARGET (https://ocg.cancer.gov/programs/target) initiative, dbGaP accession: phs000468. The data used for this analysis are available at https://portal.gdc.cancer.gov/projects. Using the University of California, Santa Cruz’s Xena platform (date accessed: March 3, 2020), we obtained mRNA-sequencing data for gene expression and clinical data for 87 of the 285 TARGET cases to determine sex differences in gene expression [21]. We identified sex differences in gene expression from 61 male and 41 female OS samples from mice with *Trp53*^*R270H*^-mutant osteoblast-specific somatic loss that underwent a *Sleeping Beauty* (SB) transposon screen [17]. Lastly, we determined sex differences in osteoblast gene expression generated by microarrays from 95 healthy individuals (53 males, 42 females; aged 40-90 years) (Gene Expression Omnibus ID: GSE15678) [18].

### Statistical analysis

From SEER, we obtained age at diagnosis (0- < 10, 10 < 20, 20-39 years), race/ethnicity (white, black, Hispanic, Asian/Pacific Islander [API]; other races excluded), year of diagnosis (2000-2005, 2006-2010, 2011-2016), primary tumor site (upper limb [topography codes: C40.0, C40.1], lower limb [topography codes: C40.2, C40.3], other [topography codes other than C40.0-C40.3]), stage of disease (local, regional, distant), and vital status (alive, dead). Chi-squared tests were performed to test for sex differences in the distribution of selected covariates. Kaplan-Meier survival curves were constructed and log-rank *p* values were utilized to compare 17-year overall survival differences between sexes.

For TARGET-OS, we used available data categorized as shown above in SEER for age, race/ethnicity, primary tumor site, and vital status along with year of diagnosis (1997-2005/2006-2014) and disease status (metastatic/non-metastatic). The 87 TARGET-OS cases did not differ by clinical characteristics from those without gene expression data (*N* = 198; results not shown) (all chi-squared *p* > 0.05).

### Gene expression analysis

#### TARGET-OS

Gene expression data as raw counts per gene was obtained. DESeq2 was used to perform differential expression testing (male–female). Heatmaps were generated (R heatmap) using the variance stabilizing transformation (VST) count data as expression values.

#### TP53-SB mutant mice

Proprietary RNA sequencing data from mouse-OS (Largaespada) was aligned using HISAT2 to a custom version of mm10 that included an additional chromosome representing the *SB* transposon. Counts per gene were generated using “Subread featurecounts” to the Ensemble v96 annotation edited to include the *SB* transposon as an additional gene feature. DESeq2 was used to perform differential expression testing (male–female). Fisher’s exact test was used to determine significant overlap between TARGET-OS and mouse-OS genes.

#### Healthy adult osteoblasts

Normalized expression and metadata from osteoblasts (GSE15678) were downloaded using “getGEO” (R GEOquery). Differential expression testing (male–female) was performed using limma. A chi-squared test was used to determine significant overlap between TARGET-OS and osteoblast genes.

### Pathway analysis

Ingenuity Pathway Analysis (IPA) and the Reactome Pathway Database [22] were used to identify biologic pathways defined by significantly differentially expressed genes by sex (adjusted *p* value < 0.05). The log2-fold change for male-female expression was entered into IPA for each gene to identify pathways that differed significantly by sex for TARGET-OS and mouse-OS (date accessed: April 25, 2020). Pathways comprised of ≥ two genes that had a significant *p* value determined by IPA were included herein. IPA BioProfiler was used to identify chemotherapies available or in clinical trials for other cancers for the TARGET-OS genes that were differentially expressed by sex. Differentially expressed gene names were also entered into the Reactome Pathway Database (date accessed: April 16, 2020). Fisher’s exact tests were used to compare overlapping IPA pathways between TARGET-OS and mouse-OS.

### LM22 analysis

CIBERSORT with the LM22 gene signature [23] was used to determine the spectrum of immune cell infiltration in TARGET-OS and mouse-OS samples. VST count data was utilized for expression. As suggested by CIBERSORT, quantile normalization was disabled, 500 permutations were run, and results are given as absolute values. Two-sided *t* tests were performed between male and female patients using LM22 immune cell type absolute values.

### Gene set enrichment analysis

Results from DESeq2 differential expression tests on TARGET-OS and mouse-OS were filtered to remove genes with N/A log-fold changes or adjusted *p* values. VST count data was used for expression values of remaining genes and written into GCT format for GSEA v4.0.3. Sex was used as the phenotypic data (male = 0, female = 1). TARGET-OS and mouse-OS were compared to the MSigDB C7 collection: immunologic signatures and 1000 permutations were performed to calculate *p* values. An alpha of 0.05 was used in all statistical tests.

## Results

### Study populations

**Fig. 1:**
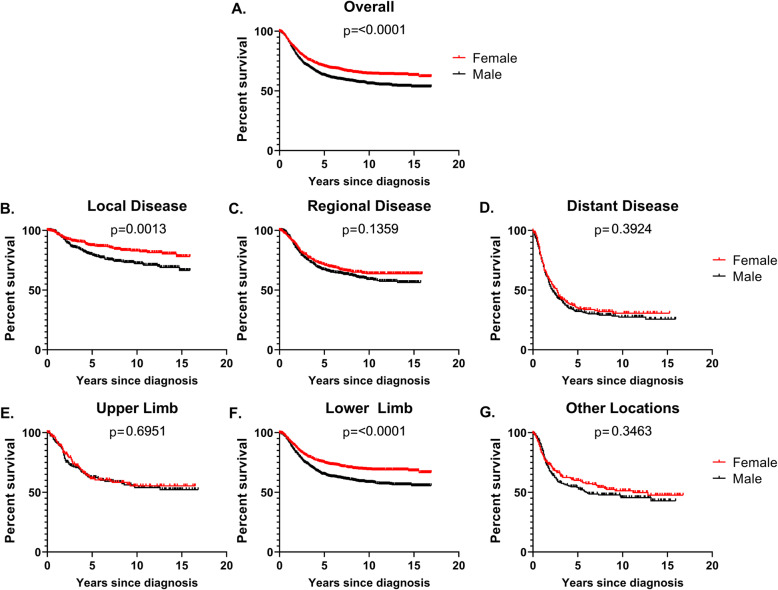
Kaplan-Meier survival curves and log-rank *p* values for sex differences in overall, long-term survival comparing males to females aged less than 40 years at diagnosis for all cases combined (**a**), by stage of disease at diagnosis (**b**-**d**) and primary tumor location (**e**-**g**), SEER 18 (2000-2016)

SEER-OS (*N* = 2940) and TARGET-OS (*N* = 87) had similar sex distributions with both approximately 55% male (Table 1). Compared to SEER-OS, TARGET-OS were more frequently aged 10- < 20 years at diagnosis (SEER 57%, TARGET 77%), non-Hispanic white (SEER 47%, TARGET 65%), had lower limb tumors (SEER 71%, TARGET 90%), and more advanced disease (SEER [distant disease] 21%, TARGET [metastatic] 25%). We observed significant 17-year overall survival differences by sex in SEER (Fig. 1). Males had inferior survival to females overall (log-rank *p* value < 0.0001), for local disease (log-rank *p* value = 0.0013), and lower limb tumors (log-rank *p* value < 0.0001). There were similar trends in 14-year overall survival in the full TARGET-OS group, but not among the cases with gene expression data (Supplemental Figure 1).

**Table 1:** Demographic and clinical characteristics of TARGET-OS and SEER-OS cases

	TARGET-OS				SEER-OS			
	Total	Females	Males	***X***^**2**^ ***p*** value	Total	Females	Males	***X***^**2**^ ***p*** value
	***N*** (%)	***N*** (%)	***N*** (%)		***N*** (%)	***N*** (%)	***N*** (%)	
**Sex**								
**Male**	50 (57.5)		50 (57.5)		1635 (55.6)		1635 (55.6)	
**Female**	37 (42.5)	37 (42.5)			1305 (44.4)	1305 (44.4)		
**Age at diagnosis (years)**								
**0- < 10**	10 (11.5)	6 (16.2)	4 (8.0)	0.06	348 (11.8)	176 (13.5)	172 (10.5)	0.03
**10- < 20**	67 (77.0)	30 (81.1)	37 (74.0)		1675 (57.0)	718 (55.0)	957 (58.5)	
**20- < 40**	10 (11.5)	1 (2.7)	9 (18.0)		917 (31.2)	411 (31.5)	506 (31.0)	
**Race/ethnicity**								
**White**	47 (65.3)	15 (46.9)	32 (80.0)	0.02	1354 (46.7)	623 (48.3)	731 (45.4)	0.20
**Hispanic**	11 (15.3)	9 (28.1)	2 (5.0)		857 (29.5)	359 (27.8)	498 (30.9)	
**Black**	7 (9.7)	4 (12.5)	3 (7.5)		459 (15.8)	211 (16.4)	248 (15.4)	
**API**	7 (9.7)	4 (12.5)	3 (7.5)		232 (8.0)	97 (7.5)	135 (8.4)	
**Missing**	15	5	10		38	15	23	
**TARGET year of diagnosis**								
**1997-2005**	34 (41.0)	12 (32.4)	22 (47.8)	0.18	-	-	-	
**2006-2014**	49 (59.0)	25 (67.6)	24 (52.2)		-	-	-	
**Missing**	4	0	4					
**SEER year of diagnosis**								
**2000-2005**	-	-	-		849 (28.9)	378 (29.0)	471 (28.8)	0.49
**2006-2010**	-	-	-		1025 (34.9)	468 (35.9)	557 (34.1)	
**2011-2016**	-	-	-		1066 (36.3)	459 (35.2)	607 (37.1)	
**Primary site**								
**Upper limb**	6 (6.9)	4 (10.8)	2 (4.0)	0.35	397 (13.5)	166 (12.7)	231 (14.1)	0.04
**Lower limb**	79 (90.8)	33 (89.2)	46 (92.0)		2081 (70.8)	910 (69.7)	1171 (71.6)	
**Other**	2 (2.3)	0 (0.0)	2 (4.0)		462 (15.7)	229 (17.6)	233 (14.3)	
**TARGET metastasis at diagnosis**								
**Non-metastatic**	65 (74.7)	25 (67.6)	40 (80.0)	0.22	-	-	-	
**Metastatic**	22 (25.3)	12 (32.4)	10 (20.0)		-	-	-	
**SEER stage of disease**								
**Local**	-	-	-		969 (36.2)	459 (38.6)	510 (34.3)	0.02
**Regional**	-	-	-		1136 (42.5)	500 (42.1)	636 (42.8)	
**Distant**	-	-	-		569 (21.3)	229 (19.3)	340 (22.9)	
**Missing**					266	117	109	
**Vital status**								
**Alive**	58 (68.2)	24 (64.9)	34 (70.8)	0.64	1971 (67.0)	921 (70.6)	1050 (64.2)	0.0003
**Deceased**	27 (31.8)	13 (35.1)	14 (29.2)		969 (33.0)	384 (29.4)	585 (35.8)	
**Missing**	2	0	2		0	0	0

### Sex differences in gene expression

**Fig. 2:**
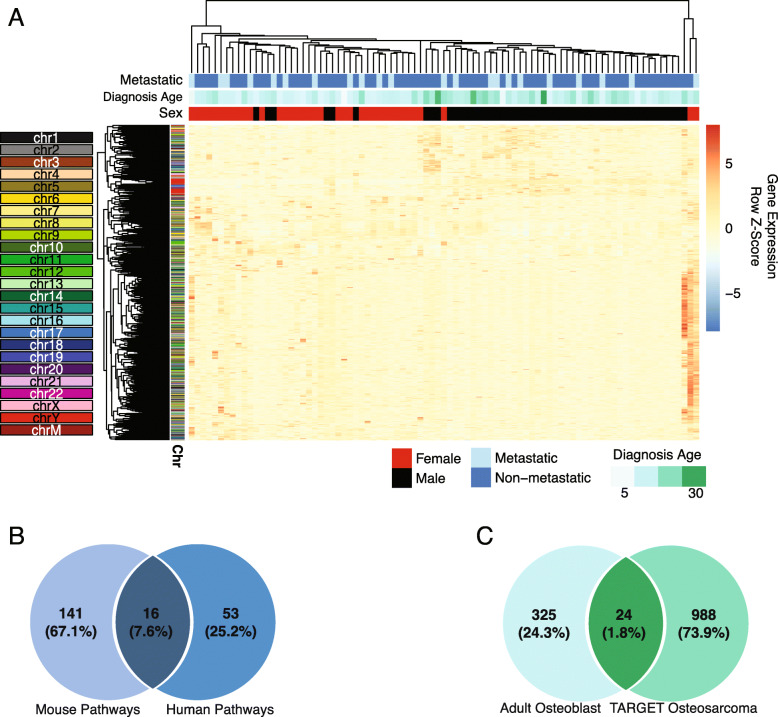
Sex differences in TARGET-OS gene expression. **a** Heatmap of all differentially expressed genes (adj. *p* value < 0.05) identified between male and female TARGET-OS samples. Top color bars indicate the sex (male = black, red = female), if metastasis was detected at diagnosis (light blue = metastatic, dark blue = non-metastatic), and age at diagnosis in years (darker green = older patients). Sample and gene order of the heatmap was dictated by unsupervised hierarchal clustering using Euclidean distance and complete clustering. Side color bars indicate the chromosomal location of the gene. **b** Overlap between enriched pathways in differentially expressed genes between male and female mouse-OS and TARGET-OS. **c** Overlap between differentially expressed genes between healthy male and female osteoblasts and TARGET-OS

We observed significant sex differences in gene expression (adjusted *p* value < 0.05) for 1018 genes in TARGET-OS (Supplemental Table 1). Of these, 98 (9.6%) were from the sex chromosomes (X *n* = 55 [5.4% of total]; Y *n* = 43 [4.2% of total]) (Supplemental Figure 2). When we performed unsupervised hierarchical clustering on all genes significantly differentially expressed by sex, clear clusters by sex emerged (Fig. 2). These clusters were largely independent of Y chromosome genes and were generally recapitulated in clustering performed without the Y (Supplemental Figure 2).

Significant sex differences in mouse-OS gene expression were observed for 918 genes (Supplemental Table 2), 723 of which had a known human ortholog. Of these, 22 (3.0%; Fisher’s exact *p* value = 0.0006) overlapped with TARGET-OS genes. Of the 22 genes that were shared between mouse-OS and TARGET-OS (Supplemental Table 5), five were on the human sex chromosomes and the remaining was autosomal. We also compared sex differences in gene expression in TARGET-OS to those in healthy adult osteoblasts [18] to ascertain baseline sex differences in osteoblast gene expression. We found 348 genes from osteoblasts that were differentially expressed by sex (Supplemental Table 3) (*n* = 39 X chromosome [11.2%], *n* = 11 Y chromosome [3.2%]), and 24 genes (6.9%; *X*^2^ *p* value = 0.017) overlapped with TARGET-OS. Differentially expressed osteoblast genes that overlapped with TARGET-OS included 11 Y, five X, and eight autosomal genes.

### Pathway analysis

Using IPA, we identified pathways that differed significantly by sex using the 1018 significantly differentially expressed genes for TARGET-OS. This resulted in 69 pathways composed of ≥ two genes that differed significantly by sex (Supplemental Table 4) out of 359 identified pathways. Top pathways were immune related including Th1 and Th2 pathways, B cell development, T helper cell differentiation, CD28 signaling in T helper cells, the complement system, T cell exhaustion signaling, various macrophage differentiation pathways, and the PD-1, PD-L1 cancer immunotherapy pathway, which is similar to the top pathway identified in Reactome, PD-1 signaling (results not shown) [22]. Regulatory pathways, including the p53 signaling pathway, also differed by sex. Using the IPA BioProfiler, we identified 24 genes significantly differentially expressed by sex that have existing therapies in use or in clinical trials for other cancers (Table 2).

**Table 2:** Ingenuity pathway analysis BioProfiler results showing genes significantly differentially expressed by sex in TARGET-OS that have existing chemotherapies for other cancers

Gene	Molecule Type	Higher gene expression	Differential gene expression, ***p*** value	Cancers known to be associated with the gene	Drug trials for particular condition associated with this gene
ACHE	Enzyme	Males	0.005	Brain tumor, leukemia, lung cancer	Phase 3
C3	Peptidase	Females	0.01	Neuroblastoma, Langerhans cell histiocytosis, leukemia	Approved, phase 2/3, phase 3
CD3D	Transmembrane receptor	Females	0.006	B cell acute lymphoblastic lymphoma, lymphoblastic B cell lymphoma	Approved, phase 2/3, phase 3
CD3E	Transmembrane receptor	Females	0.009	B cell acute lymphoblastic lymphoma, B cell acute lymphoblastic leukemia	Approved, phase 2/3, phase 3
CDK4	Kinase	Females	0.02	Breast cancer, soft tissue sarcoma, lung cancer, melanoma	Approved, phase 2/3, phase 3, phase 4
CEACAM5	Other	Females	0.04	Lung cancer	Phase 3
CHRNA7	Transmembrane receptor	Males	0.03	Bladder cancer	Phase 4
CYP19A1	Enzyme	Females	0.02	Breast cancer, fallopian tube neoplasm, ovarian carcinoma, serous peritoneal adenocarcinoma, leiomyoma, uterine cancer	Approved, phase 2/3, phase 3, phase 4
FCGR3A/FCGR3B	Transmembrane receptor	Females	0.009	Neuroblastoma, Langerhans cell histiocytosis, leukemia	Approved, phase 2/3, phase 3
FLT3	Kinase	Females	0.03	Chronic myelogenous leukemia, acute myeloid leukemia, acute myeloid leukemia associated with myelodysplastic syndrome, non-small cell lung cancer, colorectal adenocarcinoma, hepatocellular carcinoma, renal cell carcinoma, advanced stage gastrointestinal stromal tumor, pancreatic neuroendocrine tumor	Approved, phase 2/3, phase 3, phase 4
FSHR	G-protein coupled receptor	Females	0.04	Prostate cancer	Phase 3
HTR3B	Ion channel	Females	0.03	Pancreatic cancer	Phase 2/3
ICOS	Transmembrane receptor	Females	0.008	Head and neck cancers	Phase 3
IL5	Cytokine	Females	0.04	Nasal polyp	Phase 3
KCNC1	Ion channel	Males	0.0003	Prostate cancer	Phase 4
KCND2	Ion channel	Females	0.01	Prostate cancer	Phase 4
LCK	Kinase	Females	0.04	Chronic myelogenous leukemia, acute lymphoblastic leukemia, angiosarcoma, non-small cell lung carcinoma, renal cell carcinoma, soft tissue sarcoma, epithelioid malignant pleural mesothelioma, extraskeletal osteosarcoma, leiomyosarcoma, colorectal adenocarcinoma, sarcoma, malignant peripheral nerve sheath tumor, malignant soft tissue neoplasm, cancer, chondrosarcoma, ovarian cancer, colorectal adenocarcinoma, soft tissue sarcoma, melanoma, synovial sarcoma	Approved, phase 2/3, phase 3, phase 4
MUC16	Other	Females	0.03	Ovarian cancer	Phase 3
PLA2G2A	Enzyme	Females	0.03	Advanced oral squamous cell carcinoma, colorectal cancer, Langerhans cell histiocytosis, primary oral squamous cell carcinoma, soft palate squamous cell carcinoma	Phase 2/3, phase 3, phase 4
ROS1	Kinase	Females	0.03	Lung cancer, hepatocellular carcinoma, renal cell carcinoma, soft tissue sarcoma, T cell lymphoma, myofibroblastic tumor, neuroblastoma, gastrointestinal carcinoid tumor, neuroendocrine malignant tumor, pancreatic endocrine tumor, thyroid cancer, pancreatic neuroendocrine tumor, melanoma	Approved, phase 2/3, phase 3, phase 4
TGFB2	Growth factor	Males	0.04	Anaplastic astrocytoma, glioblastoma	Phase 3
TNFRSF8	Transmembrane receptor	Males	0.001	Hodgkin lymphoma, T cell lymphoma, anaplastic large cell lymphoma	Approved, phase 3, phase 4
TP53	Transcription regulator	Females	0.0002	Myelodysplastic syndrome	Phase 3
TUBB4A	Other	Males	0.02	Anaplastic glioma, anaplastic oligodendroglioma, glioma, B cell-like diffuse large B cell lymphoma, acute lymphoblastic leukemia, lymphoma, acute myeloid leukemia, non-small cell lung cancer, ovarian cancer, d breast cancer, cervical cancer, cholangiocarcinoma, endometrial carcinoma, esophageal cancer, fallopian tube cancer, follicular B cell or T cell lymphoma, gastric adenocarcinoma, pancreatic cancer, prostate cancer, sarcoma, soft tissue sarcoma, testicular carcinoma, bladder carcinoma, AIDS-related Kaposi sarcoma, anaplastic astrocytoma, anaplastic medulloblastoma, angiosarcoma	Approved, phase 2/3, phase 3, phase 4

In mouse-OS, 157 IPA pathways differed significantly by sex (Supplemental Table 5) and 16 overlapped with TARGET-OS pathways (Fisher’s *p* value = 0.03). Overlapping pathways included signaling of p53, calcium, VEGF, T cell exhaustion, and IL-4. Both TARGET-OS and mouse-OS had differences in estrogen-related pathways. Pathways were nearly identical when excluding the sex chromosomes for TARGET-OS and mouse-OS (results not shown).

### Expression measurements of immune cell composition

**Fig. 3:**
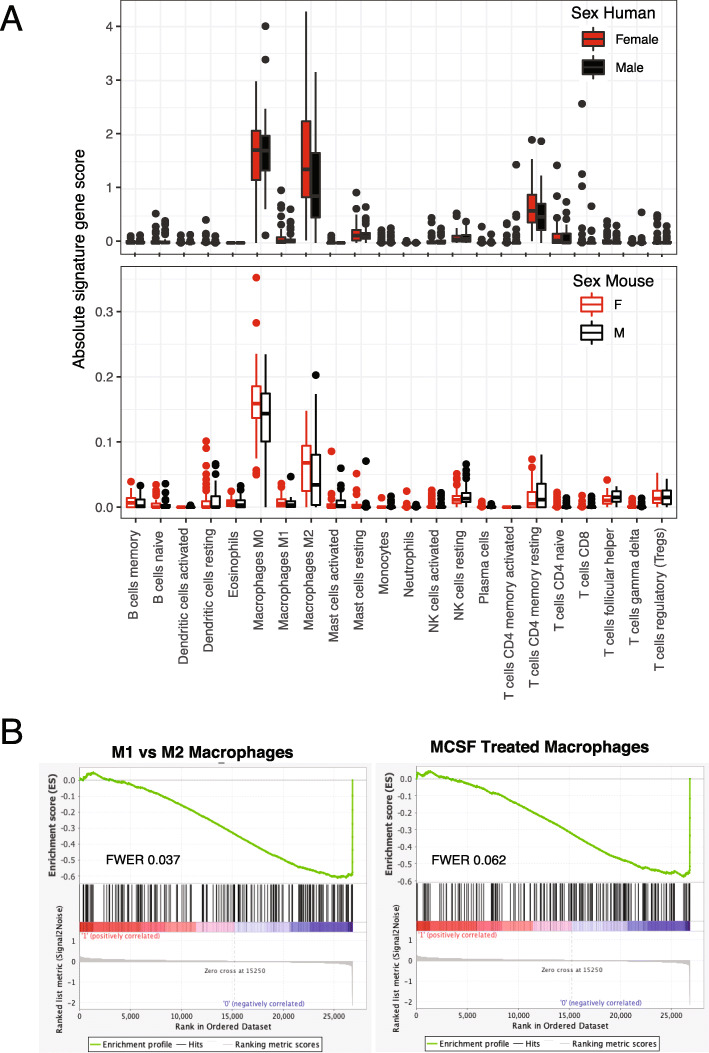
Sex differences in TARGET-OS immune cell composition. **a** Box plots of absolute gene signature scores for 22 immune cells estimated with CIBERSORT and the LM22 gene signature between male (black) and female (red) TARGET-OS (human) and mouse-OS. **b** GSEA enrichment plots and FWER values for the two most significantly enriched pathways in the MSigDB immunologic gene sets. Left plot represents the running enrichment score against GSE5099_CLASSICAL_M1_VS_ALTERNATIVE_M2_MACROPHAGE_DN and right plot against GSE5099_DAY3_VS_DAY7_MCSF_TREATED_MACROPHAGE_DN

We applied the LM22 signature [23] to determine sex differences in leukocyte composition within TARGET-OS and mouse-OS (Fig. 3, Supplemental Table 6, and Supplemental Table 7). Trends were similar in mouse-OS and TARGET-OS, but the magnitude of LM22 cell type absolute values was lower in mouse-OS. In mouse-OS and TARGET-OS, we observed an increase in M0 macrophages for both sexes and a higher level in female mouse-OS (mouse-OS *t* test *p* value = 0.045). Resting CD4 memory T cells were elevated in both sexes and a female excess in M2 macrophages (TARGET-OS *t* test *p* value = 0.056) and resting memory CD4 T cells (TARGET-OS *t* test *p* value = 0.081) was observed. Among females, there was a suggestive, though non-significant, survival benefit of having M2 levels in the top quartile (Supplemental Figure 3). In the GSEA immunologic gene set (C7) [24] (Supplemental Table 8 and 9), few strong enrichments emerged in mouse-OS and in TARGET-OS females. In TARGET-OS males, we observed stronger enrichments for two gene sets concerning the transition of macrophages from M1 to M2 and MCSF-treated macrophages (Supplemental Table 8; FWER *p* values 0.037 and 0.062, respectively).

## Discussion

Using population-based data from OS captured in SEER, we observed significantly worse long-term survival for males compared to females overall, for local disease and among lower limb tumors. Therefore, we hypothesized that sex differences in tumor genomics may be important for disease biology and future therapy development. In 87 NCI TARGET-OS cases, we observed significant sex differences in gene expression for 1018 genes. From this list, we identified 69 biologic pathways that differed by sex and found sex differences in macrophage types and states by LM22 and GSEA, respectively. In TARGET-OS, we found significant sex differences in previously identified genes aberrantly expressed in OS including *CDK4* [12], *LCK* [16, 25], *HLA-DOA* [16], *ROS1* [16, 25], *FLT3* [16], and *TP53* [12]. We observed significant sex differences in immune pathways including those involving T cells, B cells, Th1 and Th2, and the complement system. Similarly, we identified sex differences in macrophages that may underlie observed sex differences in OS progression or severity. We identified sex differences in calcium signaling, p53 signaling, and other cell cycle control pathways. The PD-1, PD-L1 cancer immunotherapy pathway also differed by sex. Additionally, we found 24 genes that were differentially expressed by sex in TRAGET-OS that also have existing chemotherapies in use for other cancers. These findings suggest there may be opportunities to use existing therapies in a sex-specific manner to improve the outcomes for OS, which would be a welcomed advancement in a cancer with poor outcomes and little treatment progress since the mid-80s [26].

We observed significant sex differences in gene expression of genes reported to be important in OS. Specifically, *CDK4* [12], *LCK* [16, 25], *HLA-DOA* [16], *ROS1* [16, 25], FLT3 [16], and *TP53* [12]. *CDK4* is a member of the cyclin-dependent kinase (CDK) 4/6-retinoblastoma protein (Rb) pathway that is altered in many human cancers, including synovial sarcoma [27], and has been found to be focally amplified in OS [12]. We observed higher expression of *CDK4* in females in our study (male-female log2-fold change = −0.63; adjusted *p* = 0.02) and Li et al. reported higher *CDK4* expression in high-grade synovial sarcomas [27] suggesting that *CDK4* may be a sexually dimorphic marker of OS disease severity to be validated in future studies. We observed higher expression of *ROS1*, a known oncogene, in females (male-female log2-fold change = −1.79; adjusted *p* = 0.004). *ROS1* is important in lung cancer with a correlative relationship to *PD-L1* expression [28]. As we observed sex differences in both *ROS1* and the *PD-L1* pathway in our study, further work examining *ROS1* as a biomarker for initiation of *PD-L1* immunotherapy in OS may be informative. *TP53*, a tumor suppressor that is most often mutated in OS [12], was observed to have higher expression in females (male-female log2-fold change = −2.41; adjusted *p* = 2.8 × 10^−7^) in our study. The relationship between *TP53* somatic mutation and gene expression is not straightforward. In adult breast cancers, missense and deletion mutations had differential impacts on gene expression that were associated with prognoses [29]. Therefore, in OS, the relationship between *TP53* mutation and expression by sex is likely to be complex. We are unable to evaluate that in this study; however, understanding sex differences in *TP53* mutation and expression will likely be very informative regarding OS outcomes in the future.

Many immune-related pathways differed by sex in TARGET-OS. In general, there are sex differences in innate and adaptive immune responses with females having more robust responses across the lifespan demonstrated by fewer early childhood infections and a higher incidence of autoimmune disease in adulthood [30–33]. Based on our previous work examining sex differences in childhood cancer incidence and outcomes [2, 7], we hypothesized that the female immune system may contribute to their better survival, particularly for OS. Interestingly, in the present analysis of TARGET-OS, we observed many immune pathways exhibiting sex differences including those regulating macrophages, B, and T cells. Additionally, we observed sex differences in the complement system, which has been reported in lupus, Sjögren’s syndrome, and schizophrenia and is thought to impact disease development in a sexually dimorphic manner [34]. Similarly, the immune system appears to be important in the observed sex differences in OS biology as well.

Macrophages, an important immune system component with an identified role in cancer metastasis, including OS [16, 35–37], were identified as differing by sex in TARGET-OS and mouse-OS [38]. Both sexes in TARGET-OS and mouse-OS had high levels of M0 macrophages and females had higher M2 macrophage levels. M2 macrophages are thought to promote metastasis in OS and other cancers [16, 35–37]. Our finding of higher levels of M2 macrophages in females with OS in both humans and mice suggests that there may be some biologically conserved mechanisms driving the abundance of this cell type in females with OS. We observed a potential survival benefit in TARGET-OS females with high M2 levels, which is counter to the observation that M2 macrophages promote metastasis and are associated with poor survival. This points to the complicated relationship between macrophages and OS and warrants further investigation in a larger study of sex differences in OS biology and outcomes. In GSEA, we observed an enrichment of macrophage differentiation pathways in TARGET-OS males, again suggesting a sexually dimorphic role of macrophages in OS. Collectively, these findings suggest an enrichment of macrophages along with variation in macrophage activity may be important in OS sex differences in both humans and mice.

We sought to identify cancer therapies that may have a sex-specific benefit in OS as survival from OS is poor and there have been few improvements in outcomes for decades. In our study, we observed sex differences in the PD-1, PD-L1 cancer immunotherapy pathway and found 24 genes differentially expressed by sex in TARGET-OS that also have chemotherapies in use for other malignancies. *PD-L1* expression is associated with a 90% excess risk in death for OS and an elevated risk of death among males (Hazard ratio: 1.25) [39]. In vitro and in vivo studies have shown that *PD-1* blockade enhances cisplatin effectiveness and may improve outcomes in humans [40, 41]. There is much debate in the literature on sex differences in immunotherapy response with males having better outcomes than females. It is hypothesized that the female immune environment has higher baseline activity so the addition of immunotherapy does not create as large of an effect [42, 43]; however, this remains to be evaluated in the pediatric and young adult populations but should be studied to determine the potential clinical benefit of immunotherapy in OS [44].

Concerning sex-specific treatment strategies, we found sex differences in genes previously identified in OS with therapies approved or in clinical trials for other cancers including *CDK4* [12] in soft tissue sarcomas and breast cancers; *LCK* [16, 25] in sarcomas and hematologic malignancies; *ROS1* [16, 25] in soft tissue sarcomas and neuroendocrine tumors; *FLT3* [16] in hematologic malignancies and other cancers; and, *TP53*, which is commonly mutated in OS and has a drug in phase 3 trials for myelodysplastic syndrome [12]. The list of gene targets in Table 2 shows that (1) consideration of sex in future therapy trials is critical as it may offer some level of benefit for males and females with OS; and (2) that there are an array of sex-specific OS genes with therapies that could be evaluated in future animal studies and human trials. These opportunities could impact OS outcomes, which have changed little in past decades.

Using 87 cases of OS from the publicly available NCI TARGET initiative, we have examined gene expression differences between males and females using various analytic tools and identified biologic pathways and immune cell composition differences by sex that may be important in disease pathogenesis. However, our work should be interpreted in light of the following limitations. The TARGET-OS sample size is limited precluding us from conducting stratified analyses by age at diagnosis, tumor location, and metastatic disease. There is no pediatric osteoblast genomic data source to our knowledge. As such, we used adult data, which likely does not fully represent the developmental stage most appropriate for TARGET-OS cases. While we did observe sex differences in immune pathways, we are unable to follow these findings up in TARGET-OS as there are no available biospecimens for genomic assays or immunohistochemistry. Further, a population-based sample of OS biospecimens and accompanying outcome data would be necessary to more clearly determine the role of tumor biology in the observed male excess in OS deaths.

### Perspectives and significance

In population-based data, males had significantly worse overall survival than females following an OS diagnosis. The mechanisms underlying these differences may depend on tumor biology or treatment response. Using the publicly available NCI TARGET-OS data, we observed gene expression differences between males and females that allowed us to discover sex differences in an array of immune-related pathways and cell types including B cells, T cells, and macrophages. Interestingly, these differences were recapitulated in a genetically engineered mouse-OS model suggesting conservation of these sex differences across species and highlighting the utility of studying OS pathogenesis in mice as human studies are costly and challenging owing to the rarity of OS. We found 24 genes differentially expressed by sex in TARGET-OS that have therapies currently available in other cancers and may represent sex-specific therapeutic targets. Placing our findings for gene expression in light of past clinical trials, particularly those that have not succeeded, may shed light on the potential mechanisms of sex in OS treatment failure. Going forward, our findings may also be used in new clinical trials as a way to determine whether drug targets are sexually dimorphic. As we observed poor long-term survival for males diagnosed with OS among children and young adults using population-based data, developing tailored strategies to improve survival for males and females with OS is critical as we work to improve survival for all individuals with this highly fatal malignancy.

## Supplementary Information

**Additional file 1: Supplemental Figure 1.** Overall survival for A. All TARGET-OS cases with survival information and B. TARGET-OS cases with gene expression data and survival information. **Supplemental Figure 2.** Gene expression in TARGET-OS patients. A. Heatmap of all differentially expressed genes (adj. *p*-value <0.05) identified between male. **Supplemental Figure 3.** Survival differences among female TARGET-OS cases with high LM22 M2 macrophage scores. **Supplemental Table 1.** TARGET osteosarcoma gene expression in males compared to females, ranked by adjusted *p*-value. **Supplemental Table 2.** Mouse osteosarcoma gene expression in males compared to females, ranked by adjusted *p*-value (*indicates gene also found significant differentially expressed by sex in TARGET OS). **Supplemental Table 3.** Healthy adult osteoblast gene expression in males compared to females, ranked by adjusted *p*-value (GEO: GSE15678) (*indicates gene also found significant differentially expressed by sex in TARGET OS). **Supplemental Table 4.** TARGET osteosarcoma Ingenuity Pathway Analysis pathways that differed significantly by sex, ranked by *p*-value. **Supplemental Table 5.** Ingenuity Pathway Analysis in mice for pathways that differed significantly by sex, ranked by *p*-value. **Supplemental Table 6.** Results from TARGET-OS LM22 analysis. **Supplemental Table 7.** Results from mouse-OS LM22 analysis. **Supplemental Table 8.** Top 10 GSEA C7 gene sets ranked by FWER for TARGET-OS females and males. **Supplemental Table 9.** Top 10 GSEA C7 gene sets ranked by FWER for mouse-OS females and males.
